# Trauma nurses' experience of repositioning practice for trauma patients: a qualitative descriptive study

**DOI:** 10.15649/cuidarte.4324

**Published:** 2025-09-01

**Authors:** Abdulkareem S. Iblasi, Yupin Aungsuroch, I Gede Juanamasta, Ghassan Watffa, Yousef Al Omran, Batla Al Shammari

**Affiliations:** 1 Assistant Professor, College of Health Science – Nursing Department, Buraimi University, Al Buraimi, Oman. E-mail: rn.iblasi@gmail.com Abdulkareem@uob.edu.om Buraimi University Buraimi Oman Abdulkareem@uob.edu.om; 2 Associate Professor, Faculty of Nursing, Chulalongkorn University Bangkok, Thailand. E-mail: yaungsuroch@gmail.com Chulalongkorn University Bangkok Bangkok Thailand yaungsuroch@gmail.com; 3 Nursing Program, STIKes Wira Medika Bali, Indonesia. E-mail: juana.masta.90@gmail.com STIKes Wira Medika Bali Bali Indonesia juana.masta.90@gmail.com; 4 Geriatric consultant & Director of Performance and Outcomes , King Saud Medical City, Riyadh First Health Cluster, Riyadh, Saudi Arabia. E-mail: gw.watfa@ksmc.med.sa King Saud Medical City, Riyadh First Health Cluster Riyadh Saudi Arabia gw.watfa@ksmc.med.sa; 5 Family Medicine Consultant & Chief Organizational Development Officer, King Saud Medical City, Riyadh, Saudi Arabia. E-mail: y.alomran@ksmc.med.sa King Saud Medical City Riyadh Saudi Arabia y.alomran@ksmc.med.sa; 6 Associated Nursing Officer, King Saud Medical City, Riyadh, Saudi Arabia. E-mail: ba.alshammari@ksmc.med.sa King Saud Medical City Riyadh Saudi Arabia ba.alshammari@ksmc.med.sa

**Keywords:** Emergency Services, Hospitals, Intensive Care Units, Nurse Administrators, Policy, Pressure Ulcer, Servicios de Emergencia, Hospitales, Unidades de Cuidados Intensivos, Administradores de Enfermería, Políticas, Úlceras por Presión, Serviços de Emergência, Hospitais, Unidades de Terapia Intensiva, Administradores de Enfermagem, Política, Úlcera por Pressão

## Abstract

**Introduction:**

Clinical evidence indicates that the low frequency of repositioning among trauma nurses contributes to pressure ulcers during hospitalization.

**Objective:**

This study aimed to understand how trauma nurses address the need for repositioning patients in an emergency room and intensive care unit in Saudi Arabia.

**Materials and Methods:**

A qualitative descriptive design was employed, and the study was reported in accordance with COREQ guidelines and checklist. Semi-structured interviews were conducted with nurses to explore how they interact with the need for repositioning patients. Rigor was ensured using the criteria established by Lincoln and Guba.

**Results:**

Fifteen nurses from a large government hospital in Saudi Arabia participated in the study. The findings revealed that the policy's clarity, the presence of teamwork, and the level of managerial follow-up influenced nurses' decisions to proceed and perform repositioning. This procedure is often omitted due to delays in medical decision-making and workload. After deciding to proceed, factors such as nursing skill and the availability of the equipment influence repositioning practice.

**Discussion:**

Clear policies, timely medical decisions, teamwork, manageable workloads, and managerial follow-up are critical in nurses’ decisions to perform or delay patient repositioning. After deciding to proceed, nurses face a second critical step: assessing their abilities, equipment availability, and the patient’s needs. Even with the intent to reposition, perceived skill gaps and inadequate equipment can significantly reduce the likelihood of completing the procedure.

**Conclusion:**

Hospitals should support the processes by improving policies and the care system for trauma patients. Repositioning is the cornerstone of pressure ulcer prevention among trauma patients; therefore, work system-level changes are needed to ensure compliance.

## Resumen

**Introducción:**

La evidencia clínica indica que la baja frecuencia con que las enfermeras de traumatología realizan el cambio de posición de los pacientes contribuye a la aparición de úlceras por presión durante la hospitalización.

**Objetivo:**

Este estudio buscó comprender cómo las enfermeras de traumatología abordan la necesidad de cambiar deposición a los pacientes en el servicio de urgencias y en la unidad de cuidados intensivos en Arabia Saudita.

**Materiales y métodos:**

En este estudio, se empleó un diseño cualitativo descriptivo de acordó con los lineamientos del COREQ y su lista de chequeo para reportar los resultados del estudio. Se realizaron entrevistas semiestructuradas a enfermeras enfocadas en explorar su interacción con la necesidad de cambiar la posición de los pacientes. La investigación aplicó los criterios de rigor establecidos por Lincoln y Guba.

**Resultados:**

Participaron quince enfermeras de un hospital gubernamental grande en Arabia Saudita. Los hallazgos mostraron que la claridad de las políticas institucionales, el trabajo en equipo y el nivel de supervisión por parte de la dirección influyen en la decisión de realizar el cambio de posición. Este procedimiento suele omitirse debido al retraso en las decisiones médicas y a la carga laboral. Además, una vez tomada la decisión de realizar el cambio de posición, factores como la habilidad de la enfermera y la disponibilidad de equipos influyen en la ejecución del cambio.

**Discusión:**

La existencia de políticas claras, la toma oportuna de decisiones médicas, el trabajo en equipo, una carga de trabajo manejable y el seguimiento por parte de la dirección son factores críticos que inciden en la decisión de realizar o posponer un cambio de posición. Una vez que se decide llevarlo a cabo, las enfermeras enfrentan un segundo momento crítico: evaluar sus propias habilidades, el equipo disponible y las necesidades del paciente. Incluso cuando se opta por cambiar la posición del paciente, las limitaciones percibidas en las destrezas y un equipo inadecuado pueden reducir significativamente la probabilidad de completar el procedimiento.

**Conclusiones:**

Los hospitales pueden apoyar los procesos mediante mejoras de las políticas y el sistema de atención de los pacientes de traumatología. El cambio de posición representa la piedra angular en la prevención de las úlceras por presión en pacientes de traumatología y es necesario introducir cambios en el sistema de trabajo para lograr un mayor cumplimiento.

## Resumo

**Introdução:**

Evidências clínicas mostram que a baixa prática de reposicionamento entre enfermeiros de trauma causa úlceras de pressão durante a hospitalização.

**Objetivo:**

Este estudo teve como objetivo compreender como enfermeiros de trauma interagem com a necessidade da prática de reposicionamento em pronto-socorro e unidade de terapia intensiva na Arábia Saudita.

**Materiais e métodos:**

Este estudo utilizou um delineamento descritivo qualitativo e as diretrizes e a lista de verificação do COREQ foram utilizadas para relatar este estudo. Os enfermeiros participantes foram entrevistados utilizando um protocolo semiestruturado que explorou como os enfermeiros interagem com a necessidade da prática de reposicionamento. Esta pesquisa utilizou os critérios de rigor estabelecidos por Lincoln e Guba.

**Resultados:**

Quinze enfermeiros de um grande hospital público na Arábia Saudita participaram do estudo. Os resultados revelaram que a clareza da política, a presença do trabalho em equipe e o nível de acompanhamento gerencial influenciam a decisão de prosseguir com o reposicionamento. Essa decisão é suprimida pela demora na decisão médica e pela carga de trabalho. Além disso, o desempenho após a decisão é influenciado pela habilidade da enfermagem e pela disponibilidade dos equipamentos.

**Discussão:**

Políticas claras, decisões médicas oportunas, trabalho em equipe, cargas de trabalho gerenciáveis e acompanhamento gerencial são cruciais para a tomada de decisões dos enfermeiros sobre a realização ou adiamento de práticas de reposicionamento. Após a decisão de prosseguir, os enfermeiros enfrentam uma segunda etapa crucial: avaliar suas habilidades, equipamentos e as necessidades do paciente. Mesmo ao optar pelo reposicionamento, a percepção de lacunas de habilidades e equipamentos inadequados pode reduzir significativamente a probabilidade de conclusão do procedimento.

**Conclusões:**

Os hospitais poderiam apoiar os processos, implementando melhorias adicionais nas políticas e no sistema de atendimento a pacientes vítimas de trauma. A prática de reposicionamento é a base da prevenção de úlceras por pressão em pacientes vítimas de trauma, e há necessidade de mudanças no sistema de trabalho para maior adesão.

## Introduction

The World Health Organization (WHO) has reported that Saudi Arabia has one of the highest prevalence of motor vehicle accidents (MVAs), with around 27 deaths per 100,000 people annually^1^. Combined with a high number of construction- and labor-related injuries,^2^ this result makes the Saudi Arabian healthcare system deal with a high prevalence of trauma patients on a daily basis^3^. The majority of these injuries require hospitalization^4^. 

Although most trauma patients are young and healthy, they are more likely to develop pressure ulcers during their hospitalization due to conditions or therapies that limit mobility^3^,^5^. However, research shows that not all immobile patients develop pressure ulcers. For instance, following hip replacement or spinal surgery, patients often require restricted mobility, but they are less likely to develop pressure ulcers^6^-^8^. According to internal statistics, 15 to 17 patients in trauma units develop new pressure ulcers each month, with a reported prevalence rate of at least 15% in 2018^9^.

Terms such as pressure ulcer, pressure injury, bedsore are all expressions that refer to tissue damage caused by gravitational pressure over bony prominences or from medical devices^10^-^12^. Experts have published several protocols and guidelines to prevent pressure ulcer development, including the use of pressure-relieving mattresses, prophylactic dressings^13^, improved nutritional levels, and regular repositioning^14^-^17^. These aspects are mandatory for immobilized trauma patients. 

Repositioning is essential in preventing pressure ulcers^12^. It is the most commonly used intervention among all others and demands physical and mental focus from nurses to conduct it^18^. Stabilizing the patient in the new position is part of repositioning^19^,^20^. It is important to avoid muscular injuries and physical strain during patient handling. Several approaches have been researched to ensure the safety of both nurses and patients during repositioning^21^. Improper techniques may put nurses and patients at risk^22^. However, inadequate repositioning procedures are common among this patient group, with hospital compliance indicators falling below 60.00%^9^.

This study aims to understand how trauma nurses perceive and implement repositioning practices among trauma patients in the emergency room and intensive care units at one of the largest trauma centers in Saudi Arabia. Currently, there are no studies available that examine nurses' actual behaviors and approaches to repositioning in trauma care, and how these may differ from those used in non-trauma conditions.

## Materials and Methods

**Study design**

The study employed a qualitative descriptive design, using semi-structured interviews, and was reported following the Consolidated Criteria for Reporting Qualitative Research (COREQ)^23^,^24^. The study aims to describe how nurses address the need for repositioning practices among immobilized trauma patients. 

**Setting**

The study was conducted at the largest government hospital in Saudi Arabia. The hospital employs approximately 5,000 nurses distributed across all categories of healthcare services. Approximately 450 nurses regularly work with trauma patients in this hospital, which has around 120 beds with an occupancy rate exceeding 90% throughout the year, all due to trauma injuries^9^. The hospital's official language is English, which was used for the interviews, as well as for the data analysis and interpretation in this study. 

According to hospital policy, when a pressure ulcer (or any skin injury) is identified, nurses must report the case to the wound care unit for assessment and treatment. If the injury is confirmed as a pressure ulcer, the wound care unit notifies the nursing quality team for documentation. For each case, the wound care unit registers the patient’s demographic information and the identity of the reporting nurse. In the current setting, several policies are in place to encourage nurses to report skin injuries observed in patients, and no blame is assigned for such reports.

**Participants and Sampling**

**Inclusion Criteria.** Eligible participants were clinical nurses currently working in the trauma emergency unit or the trauma intensive care unit who had reported at least one case of hospital-acquired pressure ulcer, based on wound care records, during the past 90-day period preceding the study's initiation (July 1–September 29, 2019). The rationale was that nurses who had reported pressure ulcer cases were likely more aware of the issue and committed to its prevention. Since repositioning is part of pressure ulcer prevention, these nurses were expected to provide insights into how such practice is implemented. Frontline nurse managers were excluded from the study as they are less likely to be directly involved in decision-making regarding repositioning practices, even though they may occasionally file reports on pressure ulcers.

**Research team.** It comprised seven members, four of whom were affiliated with the hospital. The lead researcher is the hospital's wound care manager and lead author of the study. Two team members are also family medicine consultants (at a community center) and work together. The fourth research member is a nurse recruiter. None of the team members holds administrative or managerial roles in the trauma units or supervises nurses working in trauma units. Their positions are known among trauma nurses at the same institution. The remaining three researchers are affiliated with an institution in Thailand and have no direct or indirect ties to the study institution. The research team held online meetings to monitor the study's progress and conclusions. The research team agreed that data saturation would be defined as three consecutive interviews yielding no new information^24^. 

**Sampling. **Researchers sent an email to all eligible nurses based on wound care records, which showed 42 nurses. The email explained the purpose of the study and confirmed that ethical approval had been obtained from the hospital. The email also requested a reply if they agree to participate^25^. Twenty-three nurses responded affirmatively. Participants were selected in the order of their responses, and interviews were conducted until fifteen nurses had been interviewed, at which point data saturation was reached, based on the study assumptions. 

**Data collection**

The research team developed a semi-structured interview guide based on Moerman's^26^ recommendations. All team members approved the interview items, which are presented in Table 1. The interview guide was validated by two experts in qualitative studies, and a pilot interview was conducted with the trauma nurse educator. This pilot interview was excluded from the analysis because the educator did not meet the inclusion criteria, and the purpose of the interview was solely to assess clarity and flow. No significant changes were made to the interview guide following the pilot test.

**Table 1:** Semi-structured interview guide

Phase	Items	Tips and notes
Introduction	We want to ensure that we accurately capture everything you say, so we are going to record this interview. The recording will help us remember everything you have said without any mistakes. Every word you say is important for us to understand the topic of the study. Be sure that only the research team will listen to this recording, and all recordings will be deleted upon completion of the study. We would like to ask your permission to record this interview.	
Warm-up phase	How would you describe your experience with pressure ulcer issues in your unit? What about repositioning practices?	Competency, experience, training, and understanding of nurses' empathy. Focus on facts and situation descriptions.
Core	- Why do you think you perform repositioning for some patients in trauma units? - Why do you think some nurses do not perform repositioning? - What do you think makes them do that? - How do they come to that result?	Difficult part Explore the situation Process thinking
Wrap-up	- What do you think you and other nurses need? - What should be the nature of repositioning?	Less sensitive topics
Closing	- Is there anything you would like to add? - Thanks for your participation	Closing the discussion

Interviews were conducted by the lead researcher in a private room located within the hospital, away from the trauma units. On the day of each interview, the lead researcher requests an official leave from the nurse manager of the nurse's unit. The researcher arranged seating accommodations in the room and offered hot or cold beverages based on participants’ preferences, as well as bottled water. Except for one interview conducted in the afternoon (1:20 p.m.), all interviews were held in the early morning (between 7:15 a.m. and 8:15 a.m.), as this was the most convenient time for participants. All participants consented to audio recording. The interviews were conducted between October and November 2019. No financial incentives were offered for participation. All interviews were conducted in private, with only the participant and the lead researcher present in the room. The duration of the interviews ranged from 30 to 55 minutes, as detailed in Table 2 alongside participants' demographic data. No signs of discomfort or stress were observed among the participants; all appeared relaxed and engaged in the discussion. 

**Data Analysis**

The audio recordings were transcribed into Word documents by research team members and independently verified by the lead researcher and other team members. All members reviewed the recordings and cross-checked them against the written transcripts to ensure accuracy. 

Data analysis was conducted using a thematic analysis approach, which followed four main steps^27^,^28^. First, themes were independently generated for each interview. Three research team members individually read the transcripts and identified recurring themes based on their interpretation of the data. Second, themes were compared between pairs of interviews. Each pair of transcripts was independently analyzed by the researchers. Third, themes from each interview were compared with the themes generated by the three researchers from the same interview^27^. Fourth, all themes were compared together for all interviews. The data collected are available for free access and consultation in Mendeley Data^29^.

**Rigor**

This research employed the rigor criteria established by Lincoln and Guba. Credibility was addressed by thoroughly documenting each participant's statements in full and without alteration, thereby enhancing the study's descriptive validity. Through this interpretation, the researchers deepened their understanding and analysis of the expressions and thematic clusters, further strengthening the validity of the findings. For data triangulation, the researcher cross-examined the information provided by each participating nurse. To improve the verification of interviews, each participant was consulted regarding the transcript of their interview. Meanwhile, the researchers ensured that all collected data were verifiable, thereby supporting dependability. Comparable outcomes may be achieved by replicating this study. The researchers conducted a confirmability test guided by objectivity, ensuring that the findings reflected the participants' perspectives. To further ensure trustworthiness of the results, the researchers triangulated data from multiple sources and conducted member database check-ins to support transferability.

**Ethical Considerations**

Ethical approval was obtained from the Institutional Research Board of King Saud Medical City prior to data collection (Ref: H1RI-03-Dec18-01). This study is a part of a broader study titled “The Scale Development of Nursing Repositioning Practice for Bedridden Patients in Saudi Arabia.” All nurses involved in this study agreed to participate in interviews and were informed of their right to withdraw from the study at any time. The participants provided informed written consent before the interviews, following hospital protocol^30^. 

## Results

Fifteen nurses were interviewed: eight from the trauma emergency unit and seven from the trauma intensive care unit. Participants' demographic characteristics are summarized in Table 2. The findings indicate that nurses go through two critical decision points before performing repositioning, as described below. No notable differences were observed in responses based on gender, experience, or nationality. 

**Table 2:** Participants' demographic data

Variable	n=15 % (n) /Mean ± SD
Age years (SD)	31.86 ± 6.32
Sex	
Female	86.00 (13)
Male	14.00 (2)
Years of experience (SD)	9.8 ± 6.32
Years in current unit (SD)	4 ± 1.6
Nationality	
Saudi	60.00 (9)
Indian	20.00 (3)
Filipino	6.67 (1)
Jordanian	6.67 (1)
British	6.67 (1)
Unit	
Trauma Emergency	53.33 (8)
Non-Trauma Emergency	46.67 (7)
Qualification	
BSN	80.00 (12)
Diploma	20.00 (3)

*SD: standard deviation BSN: Bachelor of Science in Nursing

**From realization to decision**

There was consensus among the participants that repositioning is important for preventing pressure ulcers in immobile patients, whether due to trauma or other medical conditions. Participants agreed that nurses can determine when a patient needs repositioning and describe this as a basic nursing skill. However, participants indicated that after recognizing the need for repositioning, nurses often encountered differences related to external factors. These included the following: 

**Repositioning policy for trauma patients**

Although the hospital has a repositioning policy for immobile patients, it lacks specific guidelines for trauma patients, a distinction that sets trauma care and trauma units apart. Most participants said that unclear instructions influenced their decision to proceed with repositioning, whether to perform it as part of routine care or to avoid it due to concerns about patient safety. Participants reported feeling less confident and more stressed when planning repositioning, as the policy does not directly provide trauma-specific guidance. Despite this, the participants clearly relied on institutional policy.

*“We know from the American Heart Association's BLS [Basic Life Support] guidelines …to not extend the neck if there's trauma, so I should follow that as the policy we have doesn't say anything specific about trauma patients when it comes to repositioning, right?” Participant 5*

*“Nurses feel safer when there's a clear policy guiding their actions.” Participant 8*

*“…repositioning should be done according to a specific protocol that tell us how and when to turn a trauma patient, but we just don't have one.” Participant 7*

**Delayed medical decisions**

Medical decisions in trauma care often depend on the results of several diagnostic examinations before a final treatment plan can be established. These evaluations may take several hours to complete all required radiological investigations. However, the participants identified the delay in decision-making as a demotivating factor when it comes to initiating repositioning, as reflected in the following quotes:

*“…they [doctors] keep doing their rounds over and over, and there's still no final decision. Is there a spinal cord injury or not? Is it safe to reposition or not? So we just can't take that responsibility! It could make the injury worse.” Participant 4*

*“We [nurses] need to wait for three or four different teams to come and tell us what to do, or what not to do, and there's no time limit for when the doctors have to make that decision.” Participant 3*

* “Nurses can't take the risk until they get the okay from the doctors. Sometimes, that takes a whole shift [a shift equals 12 hours in trauma units] or even longer.” Participant 6*

**Teamwork spirit**

Repositioning is a task that requires the collaboration of two or more nurses. Thus, teamwork plays a significant role in its successful execution. Participants explained that having a cooperative and supportive team facilitates repositioning for nurses. When one nurse requests assistance, others are willing to assist, which promotes compliance.

*“Many things enhance repositioning, like teamwork. When you ask me to help you reposition your patient, then later you’ll help with mine…” Participant 4*

**Workload**

Nurses reported that they are more likely to perform patient repositioning when the workload is manageable. However, nurses are less likely to perform patient repositioning when the workload is perceived as high. This concern was raised by four participants, suggesting the need for further investigation into this point for a deeper understanding of how nurses prioritize nursing tasks and where repositioning falls among their other competing tasks.

*“When there’s too much work going on, you end up forgetting about repositioning…” Participant 9*

* “The workload, sir… when there’s a high workload, no one's available to help with repositioning.” Participant 11*

**Managerial follow-up**

Participants agreed on the important role of head nurses in following up on repositioning practice. Nurses are often subjected to multiple distractions. The participants felt that the role of the unit head nurse was to oversee nurses' compliance with nursing care protocols, including repositioning. As such, the lack of managerial oversight was perceived to have a negative impact.

* “…The head [head nurse] should keep following up nurses’ performance in all aspects of nursing care, including repositioning.” Participant 1*

* “Reminding nurses about repositioning, follow up to see if they do it, probably do it, or don't do it, and even help them…” Participant 8.*

**From the decision to behavior**

Even after deciding to perform repositioning, nurses will encounter additional challenges that prevent them from proceeding with repositioning. Some barriers identified are the following: 

**Repositioning equipment**

Although the participants agreed on the importance of repositioning aids, many argue that such devices are not available in sufficient quantities. As a result, when a nurse needs a device that is unavailable, this often leads to delays or even avoidance of the procedure.

*“Sometimes, we can't turn a patient with bilateral chest tubes. There is something that can help, like the lifting devices.” Participant 4 *

*“… there's only one lifting device. So by the time you request it and it arrives, it takes a while, and sometimes it's already taken on the fifth floor [referring to the bariatric unit], so it takes more than an hour to get here.” Participant 6 *

**Lack of required skills**

It is reasonable to expect that nurses should have the necessary skills to perform repositioning safely and effectively. However, analysis of participants' responses indicated a perceived lack of sufficient skills for repositioning patients with specific trauma-related conditions, particularly those involving tractions, fixators, or connected devices. Participants noted that this skill gap increased their stress levels when attempting to reposition patients connected with different kinds of devices. Moreover, they emphasized that skills are needed to practice repositioning safely and effectively.

*“… how to do it in a good way with many devices or tractions... so...[they] just try not do it…” Participant 4*

* “We also need further support by doing this in front of us. Someone should come and do it for me, right? Not just say 'You should put the device here or there' but [with] no actual application, it's still a problem.” Participant 12 *

* “I don't think they [nurses] have a proper kind of training. So even if they [nurses] plan to do it, when seeing the devices and connections, they [nurses] back out [of repositioning].” Participant 3*

## Discussion

**Figure 1:**
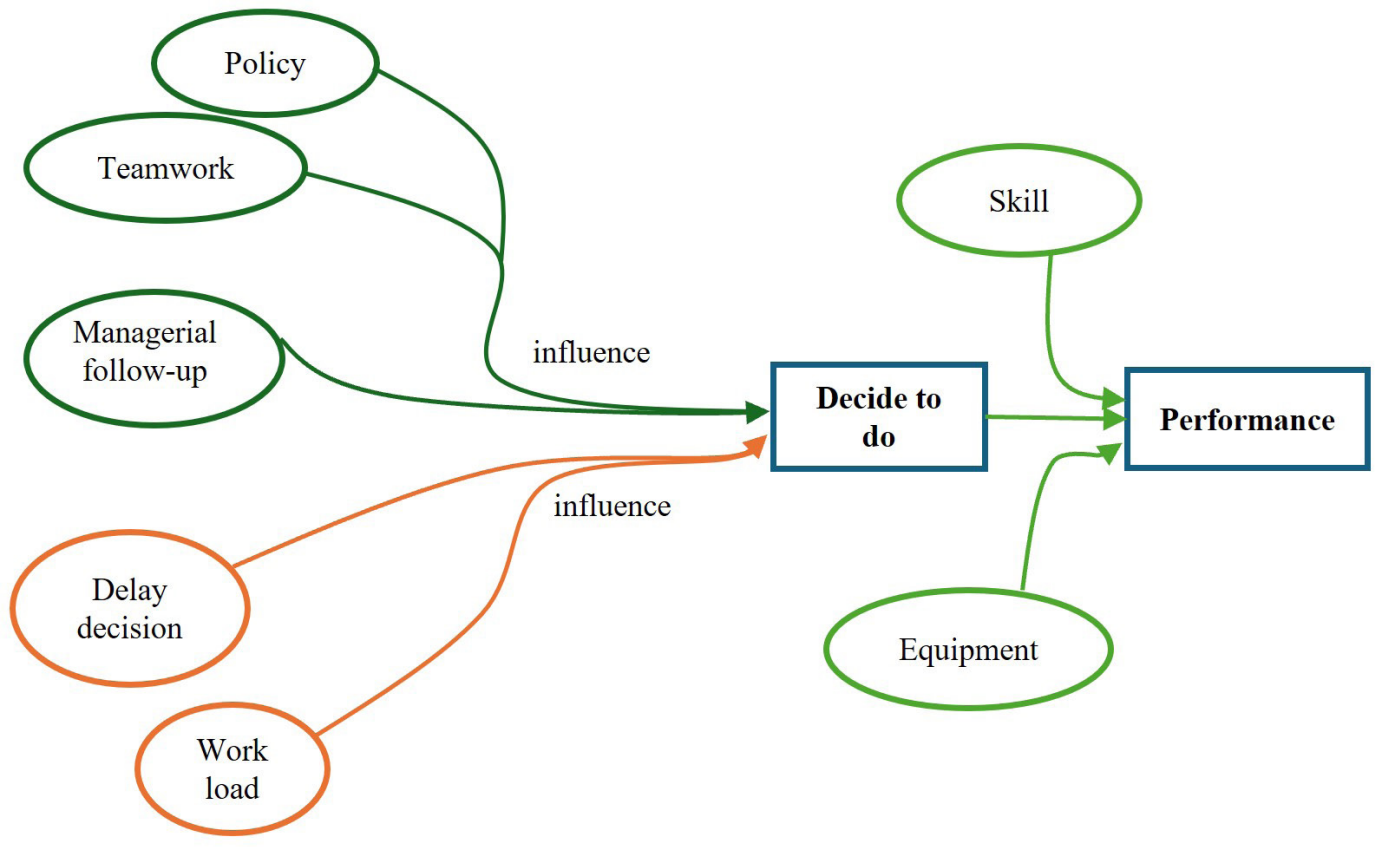
Conceptual Diagram of Repositioning Practice

Repositioning practice for trauma patients is the result of two points of interaction: the pre-decision point, when the nurse decides whether or not to reposition the patient, and the pre-behavior point, as illustrated in Figure 1. 

The presence of clear policies, the timing of medical decisions, teamwork, workload, and managerial follow-up are crucial factors influencing nurses' decisions to either perform or defer patient repositioning. The impact of delays in medical decision-making on repositioning practice was clearly a contentious issue. Although such delays are not within the scope of nursing responsibility, they significantly affect the overall quality of nursing care. Therefore, establishing a maximum timeframe for making medical decisions is necessary for repositioning interventions.

The interplay of these factors, whether proceeding with patient repositioning or not, is consistent with other studies^18^, as nurses evaluate the environment before conducting interventions. The Theory of Planned Behavior refers to this evaluation phase as behavioral intention^31^. Similarly, Orem's Theory of Self-Care describes it as nursing agency, when nurses recognize the need for care and initiate strategies to meet that need^32^. Consequently, maintaining a positive decision-making environment is crucial to promoting repositioning behavior. The results suggest that a lack of policies, delayed medical decisions, and high workload collectively reduce the possibilities of repositioning being performed. Thus, hospitals should strive to clarify their policies, improve the perceived workload, and minimize delays in medical decision-making. Hospitals should also support frontline nurse managers' responsibility in monitoring this nursing behavior. Frontline nurse managers play a crucial role in monitoring patient repositioning, which includes head nurses, charge nurses, and ward sisters^33^. 

The data further indicated that, even after deciding to perform patient repositioning, nurses face a second critical point, which is evaluating their competencies and equipment availability in relation to the patient's needs. Lifting devices help nurses place patients in a new position^34^,^35^, reduce the physical effort required to reposition them^36^,^37^, and protect nurses’ backs from injuries during repositioning^35^,^38^. However, even when nurses decide to proceed with patient repositioning, a perceived lack of skills or inadequate equipment may ultimately prevent them from performing the procedure. 

It is evident that human behavior is influenced by many individual and interconnected, complex factors; therefore, it is not always easy to understand all the relevant factors. However, gathering descriptions of patient repositioning from the participants' accounts provides valuable insight into the urgent need to modify policies and procedures related to trauma care services. Specifically, the findings highlight the urgent need to develop clear policies and protocols for repositioning different types of immobilized trauma patients, establish time limits for medical teams to clarify repositioning restrictions, ensure these timeframes align with accepted repositioning intervals in Saudi Arabia (approximately every two hours)^39^, and provide tools and equipment to support repositioning in trauma units.

**Limitations**

The current study had some limitations. First, four members of the research team were employed at the hospital where the study was conducted, which may have influenced participants' responses in some way. Second, this study was conducted in a trauma center; therefore, these results might differ if the study were replicated in other healthcare centers or discussed in different contexts. Finally, the current study explored the views of clinical nurses, excluding those in managerial or administrative roles who might have different viewpoints or perceptions of the facts. 

## Conclusions

There are two overarching explanatory themes regarding trauma patient repositioning: factors influencing the decision to perform repositioning, and factors supporting the transition from decision to action. The results highlight the challenges trauma nurses face in implementing repositioning and the need for clear policies, teamwork support, further follow-up from frontline managers, appropriate skills, and equipment. Additionally, previous studies have shown a significant correlation between pressure ulcer development and workload; however, the extent to which this relationship may differ in trauma units needs further research. Moreover, follow-up studies are required to investigate the changes in the repositioning practice and pressure ulcer prevalence among these units after modifying these factors. Finally, future studies should develop and validate tools to measure repositioning practices in trauma units. 

**Relevance for clinical practice**

Nurse managers are encouraged to use these findings to improve hospital policies related to trauma patient repositioning, clarifying indications, contraindications, patient conditions, timing, and the number of staff needed for safe implementation of repositioning. A clear and specific standard is needed for each ward. Additionally, nurse managers should promote skill development and updating in the use of repositioning devices. Their role in supervising and motivating nursing staff to perform repositioning is crucial.
